# In Nature's School

**Published:** 1889-06-29

**Authors:** 


### The Doctor's Pulpit.

### IN NATURE'S SCHOOL.

### Object Lessons.

Whoever takes the trouble to look deeply into life soon discovers that knowledge is advanced when things are understood, not when mere words are remembered. True science is practical. If this be indeed so, a ploughman may be more scientific than a Fellow of the Royal Society. Ours is a world of things. There is no need for a man to rack his brain in the endeavour to master incomprehensible hypotheses and impossible definitions. Let him look steadily and intelligently at things, and try to understand them. When he has possessed himself of their secrets, he can afford to laugh at the academic tyro who deludes himself with a conjurer's juggle of words. The human creature does not advance to any very great age before he finds out that he has a stomach which can be hungry, and a head which, when it comes into contact with a harder object than itself, can feel pain. He discovers, moreover, the nature of a larder, and of a human providence which goes by the universal name of "mother." What schoolmaster has taught him these elements of practical knowledge? Nature has been his school dame, and she has taught him well. On these topics of hunger and pain, food and mother, he can pass any reasonable examination even in his tenderest years. These object lessons have required no blackboards and mysterious algebraic formulæ in order to get themselves woven into the young human being's personality and to become a veritable part of himself. So far as it goes, his knowledge is profound; it is science indeed; it is practical.

It is curious to note how soon the very young child finds out the way to satisfy his hunger, and how to avoid pain. The care with which he does avoid pain, the trouble he takes in walking so as to escape bringing his nose into unhappy collision with the floor for example, the certainty he appears to feel that the floor will be victorious, and the candid recognition that this is inevitable and must be reckoned with—all these things are in a high degree instructive to those who look on from the exalted dignity of early adult life, or of middle or old age. "What can't be cured must be endured" is a part of infantile philosophy which is understood and practised almost at the very outset of infant life. The idea of fixedness, of certainty, of law, is one which every person needs to work into his mind and soul, into his practical intellect. This idea Nature is eminently qualified to teach. What she does is done. Not only may it be said, as in the child's rhyme-book, that "all the king's horses and all the king's men" cannot undo it, but all the kings themselves, and all the queens, and prime ministers, and judges and policemen, and generals and soldiers, cannot undo it. Men have to learn that in the world where they are placed to work out their destiny there are laws and limitations, and a fixed order beyond which none of them can pass, and within which each man must exercise his own little or great measure of freewill and practical energy according to his own choice and selection. In this hive each human bee must work, here and nowhere else; here, surrounded by other bees as truly the care of Nature as himself; here, limited by the bounds of the hive, by the dimensions of the cell, by the rights of his fellow-bees, and by the powers and capacities which are proper to creatures of his own peculiar order of existence.

The object lessons of Nature do not merely impress upon men the ideas of law and limitation. They would be things to be dreaded, and perhaps even hated, if they did. But few men hate the world in which they live, or even dread it. Notwithstanding the fixed and immovable character of Nature and her laws, there is a profound conviction in the universal mind that the world of things, if not of men, is a kindly place. The summer is genial and generous, and even the winter is not all ice and frost and stormy wind. Nature is not all granite rocks. She possesses rich soils, and corn and fruits, and many things which untutored man finds abundantly satisfying to his hunger. Civilised man, still more blessed, has discovered that industry is a key which will open the doors of her most secret hiding-places, from which he may bring forth wealth and pleasure of all kinds. These two ideas of law and Providence, of control and goodwill, of necessity and generosity, are clearly to be gathered from any honest study of Nature's appearances and operations. On these man has learned to rely with certainty. They are his directors and his rewarders from day to day and from year to year.

Just as the child finds out that a tumble on the floor will probably cause pain and injury to some part of the body, so do older people come to learn that other actions have consequences which Nature has stamped with the marked disapproval of pain, of injury and of loss. We have not to travel far to find striking object lessons which impress these facts upon us. Excess is one of the things upon which she places a firm hand, and will by no means permit, or if she permits she punishes. It need not necessarily be vicious excess. Moderation is Nature's cardinal virtue: she rewards it in a thousand ways; but excess she hates. Take, for example, two opposite poles of immoderate behaviour—immoderate religion and immoderate drinking. A man, forgetting he is mortal, and material, and human, will "try to wind himself too high for mortal man beneath the sky." He will multiply his churchgoings, fast unduly, mortify flesh and spirit, until he becomes both mentally feeble and bodily diseased, —in short, an excitable, irrational, useless, and injurious fanatic. He refuses to follow the moderate rule of Nature, and as he has discarded her, so she will forsake him. Left to his own devices, he goes from foolish to more foolish behaviour, plunges into deeper depths of childish inconsequence, and at last finds himself, as the writer has frequently seen, distracted almost to madness, and sick almost to death. Nature has allowed him the full length of his tether compatibly with sanity and life. Now she lays the strong hand of illness upon him and shuts him up, like Joseph in ward, that he may have leisure for reflection and regret. The religious man should remember that Nature is God at work. He who sees and understands her sees and understands God. Law, the law of the lawyers, is only *said to be* the "perfection of reason." Nature, which is the work of God, *is* the "perfection of reason." God will not command one thing with his voice and do another with his hand. Let the man whose religion is a harassing care to him think of that. It would be more than strange if reason, which is the perfection and safety of all other things, should be both useless and injurious in religion.

Vicious excess, such as drunkenness and other vile behaviour, is as hateful to Nature as it is to religion. The vicious man does not believe that. He will often, indeed, deceive himself with the idea that in indulging his passions he is but obeying the voice of Nature. If that be so, then every thief, every forger, every murderer, may charge upon Nature the guilt of his crime; for each one of these feels within himself, somewhere, as strong an incentive and temptation to commit his particular crime as does the drunkard or the lascivious man to indulge his passion. What has Nature plainly declared with regard to man? That he is a reasoning being, and that not impulse nor passion, but reason, must rule him. So far from it being true that he who commits vicious excess is acting according to Nature, it is the opposite of truth. For how does Nature treat him who so indulges? She punishes him with the severest of punishments—bodily punishments, mental punishments, moral punishments. Very often she destroys him utterly—first his mind and then his body. General paralysis, imbecility, insanity, death, are quite common results of vicious physical excesses. Not only is every physician familiar with this, but every experienced man has seen it at least once in his lifetime.

On the other hand, how does Nature deal with those who, in obedience to the voice of reason and prudence, control their passions and act with justice and moderation? She rewards them by increasing the strength and authority of reason and diminishing the force and imperiousness of passion. The increased energy and capacity of the reason leads to various advantages in the struggle for survival. Thus it will often be found that of two men—beginning life at the same level of station, means, education, and health—one will end in the prison or the poorhouse, with a diseased body and worse than animal's brain; the other will be surrounded by every physical comfort, and sustained by the approbation of just and discriminating men.

Now, there is no appeal to religion here. The matter is put on the purely economic ground of profit and loss. There is a school of Nature in which all men are pupils perforce, whether they will or they will not. The mistress of the school has a very distinct and clear voice, which nobody can fail to hear if he does but listen. But listen he must. If a man cannot obtain money, even the smallest coin of the realm, without effort of some kind, how does he expect to obtain learning, the very highest of all learning, the learning that makes him understand the whole universal scheme of things by which he is surrounded—how does he expect to obtain that without any effort at all? The thing does not stand to reason. Thinking, hard, steady, continuous thinking, is out of fashion. But it must be brought into fashion again, or the present and future generations will receive many a sound box on the ear from Mistress Nature, and worse punishments by far. It is Nature's delight, as it is every schoolmaster's who is not a knave, to turn out first-class honour men. She hates boobies; and why not? But, perhaps, she hates prigs still more! Nothing is more contemptible than the little self-satisfied specialist, who, because he can grind the point of a steel pen better than his neighbour who only cuts the slit, thinks himself the greatest genius born. Specialists, of course, we must necessarily have, just as we must have gardeners, and hop-pickers, and tailors. But surely, surely, he is a sorry fellow who is not able to find out that tooth-drawing, or ear-curing, can never be one of the chief industries of the world. Let the specialist, for a little time every day, come out of his corner of the school, and away from his particular blackboard, and let him share not only in the general studies of the place, but also in the humanising sports, and sometimes even in the not unwholesome fights. If we may use a schoolboy phrase, the school of Nature is a very "jolly" place, and its object lessons are sometimes as entertaining as they are practically educational if a man have but the sense to use them as they are meant to be used.

